# Cardiac Safety of Pegylated Liposomal Doxorubicin After Conventional Doxorubicin Exposure in Patients With Sarcoma and Breast Cancer

**DOI:** 10.7759/cureus.44837

**Published:** 2023-09-07

**Authors:** Maher Alhaja, Sherry Chen, Alan C Chin, Brian Schulte, Carlo S Legasto

**Affiliations:** 1 Oncology, Department of Pharmaceutical Services, University of California San Francisco, San Francisco, USA; 2 Oncology, Department of Medicine, University of California San Francisco, San Francisco, USA

**Keywords:** anthracycline lifetime cumulative dose, breast cancer, sarcoma, cardiotoxicity, pegylated doxorubicin, conventional doxorubicin

## Abstract

Background: Lifetime cumulative doses of conventional doxorubicin (>450 mg/m2) are associated with dose-dependent cardiotoxicity. In sarcoma and breast cancer, conventional doxorubicin is often utilized in the adjuvant setting, whereas pegylated liposomal doxorubicin (PLD) is typically reserved for recurrent and metastatic disease. PLD is believed to be associated with reduced cardiotoxicity compared to conventional doxorubicin. Limited data exists evaluating the cardiotoxicity associated with PLD treatment after conventional doxorubicin, especially when doxorubicin lifetime doses approach the established cumulative total lifetime dose of 450-550 mg/m2. This study aims to further qualify the cardiac safety of PLD use in patients who have had prior exposure to conventional doxorubicin.

Methods: This was a single-center, observational, retrospective cohort study conducted in patients ≥18 years with sarcoma or breast cancer who were exposed to conventional doxorubicin from an earlier line of treatment before PLD between January 2010 to May 2022. Patients were evaluated for the presence of cardiac toxicity at any point in their treatment course. Cardiac toxicity was defined as ≥ 10% decrease in left ventricle ejection fraction (LVEF) or a new diagnosis of heart failure within six months after PLD cessation. The time interval between the last conventional doxorubicin exposure and PLD initiation and the time interval between PLD initiation and LVEF monitoring were also analyzed.

Results: 494 patients were screened, and 50 met inclusion criteria: eight with sarcoma and 42 with breast cancer. The median lifetime cumulative conventional doxorubicin dose in patients with sarcoma was 450 mg/m2 with a maximum dose of 825 mg/m2 and 240 mg/m2 with a maximum dose of 300 mg/m2 in breast cancer patients. The median lifetime cumulative PLD dose was 105 mg/m2 (range: 35-150 mg/m2) in the sarcoma group and 105 mg/m2 (range: 35-510 mg/m2) in the breast cancer group. A decrease of ≥ 10% in LVEF was not observed in the sarcoma group. Patients with breast cancer had available LVEF data on PLD, and three of these patients experienced ≥ 10% in LVEF drop, with one of these patients diagnosed with heart failure. The average cumulative dose of PLD administered in patients with > 10% decrease in LVEF was 177 mg/m2 and had an average of 3.5 cycles. Five sarcoma patients initiated PLD treatment within two years after conventional doxorubicin exposure, while most breast patients initiated PLD treatment at least 10 years following conventional doxorubicin exposure. The average time from PLD initiation to first and second available LVEF monitoring was one and five months in the sarcoma group and three and eight months in the breast cancer group, respectively.

Conclusion: PLD administration in patients with prior exposure to conventional doxorubicin appears to be safe, with limited cardiotoxicity in patients with sarcoma and breast cancer. Future research is needed to determine if and how often routine cardiac monitoring is needed for patients on PLD without existing cardiac risk.

## Introduction

The existence of a dose-dependent cardiotoxicity correlating with lifetime cumulative doses of doxorubicin has been well established [1,2]. Early studies of administration of the medication evinced a possible correlation, with further study ultimately impacting prescriber guidelines and FDA labeling [3]. However, there is limited evidence on the cardiac safety of high cumulative doses of pegylated liposomal doxorubicin (PLD) in patients with sarcoma and breast cancer (BC) who have had previous exposure to conventional doxorubicin in the adjuvant setting. The purpose of this study is to evaluate the risk of cardiotoxicity associated with PLD administration in patients with prior exposure to conventional doxorubicin. Given that many patients with sarcoma and BC receive conventional doxorubicin in earlier settings and may require PLD in later lines of treatment, it is vital to assess the risk of cardiotoxicity in this population.

It is believed that doxorubicin causes cardiotoxicity due to myocyte free radical damage that is enhanced by high peak plasma levels and repetitive damage to myocyte mitochondria [4]. Encapsulating doxorubicin in a polyethylene glycol-coated liposome allows doxorubicin to maintain its efficacy while significantly reducing the risk of cardiotoxicity by minimizing systemic exposure [5]. When compared to conventional doxorubicin, PLD is associated with less toxicity to healthy tissues due to a lower plasma level of free doxorubicin and limited penetration into cardiac muscle [1,6].

The primary treatment for localized sarcoma is surgical resection. Although still debated, chemotherapy is often used as neoadjuvant or adjuvant therapy for patients with sarcoma to prevent the future occurrence of metastatic disease. Many histologic subtypes are likewise treated with conventional doxorubicin as a first-line option in the advanced disease setting [7,8]. Lifetime cumulative doses of conventional doxorubicin greater than 450 mg/m2 are associated with dose-dependent cardiotoxicity [2]. Although some recent data suggest that higher lifetime cumulative dosing of doxorubicin may be safe when co-administered with dexrazoxane, this has yet to be established in prospective phase III trials [9,10]. PLD has demonstrated itself to be a well-tolerable option with documented reduced or minimal cardiotoxicity, even in high cumulative doses [8]. The main dose-limiting side effect of PLD is palmar-plantar erythrodysesthesia (PPE). The prevalence of this side effect limits the PLD dose compared with conventional doxorubicin on a per-cycle basis [9]. PLD has been shown to be an active agent in advanced, metastatic, relapsed, or refractory sarcoma [8]. There are currently limited studies evaluating the safety of PLD with prior exposure to conventional doxorubicin in sarcoma patients.

Primary treatment for localized BC may rely on a combination of surgical resection, radiation therapy, and systemic therapy. Conventional doxorubicin may be utilized in early-stage BC for appropriate patients, whereas PLD is used in advanced and metastatic BC [11]. Prior data regarding an association between the total cumulative lifetime dose of anthracycline and subsequent PLD administration has not been conclusive for patients with BC [12,13].

A recent study evaluated the cardiac safety of PLD in metastatic BC where patients were previously treated with conventional anthracyclines [11]. About 40% of patients received doxorubicin with a mean cumulative dose of 253.1 mg/m2 (± SD 25.2), and about 60% previously received epirubicin with a mean cumulative dose of 588.0 mg/m2 (± SD 97.3). The mean cumulative PLD dose was 378.4 mg/m2 (± SD 250.2), with a range of 100 to 1200 mg/m2. Grade 2 decline in LVEF was observed in two patients (6.5%) who received cumulative PLD doses greater than 400 mg/m2. No grade 3 or 4 events were observed [14].

Another study evaluated metastatic BC patients treated with PLD after conventional doxorubicin exposure and found a non-significantly higher risk of new-onset heart failure in patients who received cumulative conventional doxorubicin doses ≥ 400mgm2 (HR 4.07; 95% CI 0.88-18.93; p = 0.07) [15]. Cumulative doses of PLD ≥ 400 mg/m2 have been shown to not increase cardiac toxicity risk in patients with gynecologic malignancies who have not been exposed to conventional doxorubicin. The mean cumulative dose in the study was 663.9 mg/m2 (range 400-1,524 mg/m2), demonstrating that PLD is tolerated at high cumulative doses with minimal increased risk of cardiac toxicity, although this remains to be validated in prospective studies [16].

PLD is widely used in the advanced setting for patients with sarcoma and BC; however, the cardiac safety of PLD after conventional doxorubicin exposure is not well documented [7,11]. Given that many patients with sarcoma and BC receive conventional doxorubicin in earlier lines of treatment and may require PLD in a later line of therapy, we aim to assess the risk of cardiotoxicity of PLD following conventional doxorubicin exposure.

## Materials and methods

Patient eligibility

In this single-center, retrospective, observational cohort study, data was reviewed between January 1, 2010, and May 31, 2022, at the University of California at San Francisco. Patients were included if they were 18 years or older with a primary diagnosis of sarcoma or BC and were treated with PLD after previous exposure to conventional doxorubicin. Patients participating in clinical trials were excluded. This study was approved by the local Institutional Review Board with a waiver for written informed consent.

Study design and outcomes

Data was obtained through electronic records and compiled using REDCap (Research Electronic Data Capture) electronic data capture tools [17]. The primary outcome was cardiotoxicity, which was defined as ≥10% decrease in left ventricle ejection fraction (LVEF) while on PLD or a new diagnosis of heart failure within six months after PLD cessation. Cardiotoxicity was evaluated in patients without LVEF data by searching chart notes for "heart failure", "cardiac events", and "cardiotoxicity". Lifetime cumulative conventional doxorubicin and PLD doses were recorded for each patient. For patients who had a >10% drop in LVEF, the time from PLD initiation to decline was measured. Data were collected based on available echocardiogram tests. Categorical variables were assessed using the chi-square test. All statistical analyses were performed using STATA 17 software.

Concurrent or prior exposure to cardiotoxic agents was collected. Commonly used cardiotoxic agents in sarcoma and BC patients collected were: ifosfamide, trastuzumab, pertuzumab, ribociclib, and pazopanib. Any use of a cardioprotective agent, specifically dexrazoxane, was also analyzed.

## Results

During the study period from January 2010 to May 2022, 494 patients receiving PLD were screened, and 50 patients met inclusion criteria: eight (16%) sarcoma and 42 (84%) BC patients. The sarcoma and BC types included can be found in Table 1. Patients were excluded if they were participating in clinical trials, had a primary cancer diagnosis other than sarcoma or BC, or were receiving PLD without prior conventional doxorubicin exposure. There were a total of 7 sarcoma types, including leiomyosarcoma (n=2, 25%), liposarcoma (n=1, 12%), osteosarcoma (n=1, 12%), Kaposi sarcoma (n=1, 12%), unclassified spindle sarcoma (n=1, 13%), rhabdomyosarcoma (n=1, 13%), and malignant peripheral nerve sheath tumor (n=1, 13%), with leiomyosarcoma being the most common type. BC receptor status was HR+/HER2- (n=29, 69%), HR-/HER2-(n=10, 24%), HR+/HER2+ (n=2, 5%), and HR-/HER2+ (n=1, 2%).

**Table 1:** Sarcoma and Breast Cancer Classification

Cancer Type	Cancer Type Classification	Number of Patients (%)
Sarcoma	Leiomyosarcoma	2 (25)
	Liposarcoma	1 (12)
	Osteosarcoma	1 (12)
	Kaposi Sarcoma	1 (12)
	Unclassified spindle sarcoma	1 (13)
	Malignant peripheral nerve sheath (MPNS)	1 (13)
	Rhabdomyosarcoma	1 (13)
Breast Cancer (BC)	HR+/Her2-	29 (69)
	Triple negative	10 (24)
	HR+/Her2+	2 (5)
	HR-/Her2+	1 (2)

Baseline demographics are displayed in Table 2. The mean age at the time of PLD initiation in the sarcoma and breast cohorts was 55 years and 54 years, respectively. Fifty percent of patients in the sarcoma group were female (50%), and most patients in the BC group were female (98%). All sarcoma patients were Caucasian, while the BC group included Caucasian (76%), Asian (15%), Black (2%), Other (1%), and Unknown (5%). One patient in the sarcoma group and none of the patients in the BC group had a past medical history of heart failure. The median lifetime cumulative conventional doxorubicin dose was higher in sarcoma patients (450 mg/m2 with a maximum dose of 825 mg/m2) compared to breast patients (240 mg/m2 with a maximum dose of 300 mg/m2). Dexrazoxane administration during conventional doxorubicin treatment occurred in three sarcoma patients (38%) and none of the BC patients. Prior or concurrent exposure to cardiotoxic agents while on PLD was observed in three sarcoma patients and in four BC patients. These agents included ifosfamide, pazopanib, trastuzumab, pertuzumab, everolimus, and ribociclib.

**Table 2:** Baseline Demographics Other*: pertuzumab, sunitinib, sorafenib, lenvatinib, vandetanib, osimertinib, afatinib, lapatinib, tucatinib, neratinib, ribociclib, and pazopanib. Any use of cardioprotective agent, specifically dexrazoxane, was also analyzed. PLD: Pegylated liposomal doxorubicin

	Soft Tissue Sarcoma (8 Patients)	Breast Cancer (BC) (42 Patients)
Age (year) (Mean ± SD)	55 ± 18	54 ± 12
Sex, Females	4 (50%)	41 (98%)
Race		
White	8 (100%)	32 (76%)
Asian	-	6 (15%)
Black	-	1 (2%)
Other	-	1 (2%)
Declined	-	2 (5%)
PMH of Heart Failure Prior to PLD Initiation	1 (13%)	None
Lifetime Cumulative Conventional Doxorubicin Dose (mg/m2) (Median, range)	450 (75 to 825)	240 (50 to 300)
Dexrazoxane Administration while on Conventional Doxorubicin	3 (38%)	None
Prior or Current Exposure to Cardiotoxic agents While on PLD (Ifosfamide, Epirubicin, Trastuzumab, Other*)	3 (38%)	4 (10%)

**Figure 1:**
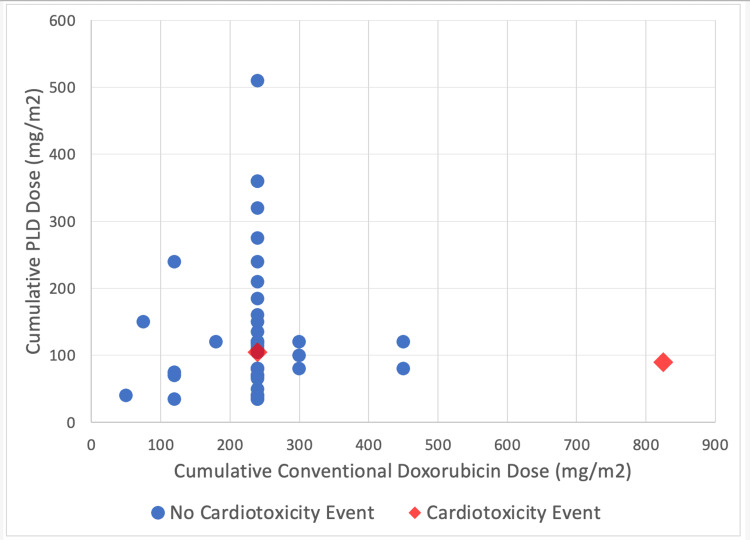
Cardiotoxicity Associated With Cumulative Doxorubicin and PLD Dose

The median lifetime cumulative PLD dose in patients with sarcoma and BC was 105 mg/m2 in both groups, with a maximum of 150 mg/m2 and 510 mg/m2, respectively (Table 3). The mean baseline LVEF before PLD initiation was 60% and 63% in sarcoma patients and BC patients, respectively. None of the sarcoma and BC patients received dexrazoxane while on PLD. Of the 42 patients in the BC group, 22 had available echocardiogram results while receiving PLD, and three patients experienced a 10% or greater decline in LVEF. The mean time to LVEF decline from PLD initiation was five months in these patients. The mean cumulative PLD dose in BC patients with a >10% decrease in LVEF was 177 mg/m2. One patient in the BC group developed heart failure within six months after stopping PLD. A LVEF decrease of 10% or more was not observed in the sarcoma group. One sarcoma patient had a previous diagnosis of heart failure prior to PLD initiation. Patient-specific lifetime cumulative dosing of conventional doxorubicin and PLD and if the patient experienced a cardiotoxicity event is shown in Figure 1.

**Table 3:** Primary Outcomes PLD: Pegylated liposomal doxorubicin, LVEF: Left ventricle ejection fraction

	Soft Tissue Sarcoma (n = 8)	Breast Cancer (BC) (n = 42)
Lifetime Cumulative PLD Dose (mg/m2) (Median, range)	105 (35 to 150)	105 (35 to 510)
LVEF (%) Before PLD Initiation (Mean ± SD)	60 ± 5	63 ± 5
Dexrazoxane Administration while on PLD	None	None
> 10% Decrease in LVEF while on PLD n (%)	None	3 (7%)
Time from PLD Initiation to ≥10% Decrease in LVEF, Months (Mean ± SD)	-	5 ± 3
Cumulative PLD Dose in patients with ≥10% Decrease in LVEF, mg/m2 (Mean ± SD)	None	177 ± 88

The time between PLD initiation and conventional doxorubicin cessation in sarcoma patients was mostly between 0 and 2 years (63%) and was mostly greater than 10 years (31%) in the BC group (Table 4). The average time from PLD initiation to the first and second available LVEF was one and five months in the sarcoma group, respectively. The average time from PLD initiation to first, second, third, and fourth available LVEF in BC patients was three, eight, nine, and 14 months, respectively.

**Table 4:** Secondary Outcomes PLD: Pegylated liposomal doxorubicin, LVEF: Left ventricle ejection fraction

	Soft Tissue Sarcoma (n = 8)	Breast Cancer (BC) (n = 42)
Time between PLD Initiation and Conventional Doxorubicin Cessation		
0-2 years	5 (63%)	7 (17%)
3-5 years	1 (13%)	11 (26%)
6-7 years	-	4 (10%)
8-10 years	1 (13%)	3 (7%)
>10 years	1 (13%)	13 (31%)
Unknown	-	4 (10%)
Time from PLD Initiation to First Available LVEF, Months (Mean ± SD)	1 ± 2	3 ± 3
Time from PLD Initiation to Second Available LVEF, Months (Mean ± SD)	5 ± 4	8 ± 4
Time from PLD Initiation to Third Available LVEF, Months (Mean ± SD)	-	9 ± 1
Time from PLD Initiation to Fourth Available LVEF, Months (Mean ± SD)	-	14 ± 4

## Discussion

Limited data exists evaluating the cardiac safety of PLD in sarcoma and BC patients following previous exposure to conventional doxorubicin. Our results do not appear to suggest an increased incidence of cardiotoxicity in these patients. Among the 50 patients included in this study, three patients with BC experienced ≥ 10% decrease in LVEF, but none in the sarcoma group, despite the higher lifetime cumulative doxorubicin dose. The small sarcoma sample size (N=8) and the receipt of dexrazoxane during conventional doxorubicin (38%) may have contributed to no observations of ≥ 10% decrease in LVEF. It is possible that other external factors have a bearing on the incidence of cardiotoxicity, such as the history of administered medications and prior radiation exposure. The most common cardiotoxic agents used in the BC group were trastuzumab, pertuzumab, and ribociclib. Among the three patients with BC who had a greater than 10% decrease in LVEF, only one patient developed clinical manifestations of heart failure within six months of PLD cessation. Notably, this patient had a history of prior radiation within six months of heart failure diagnosis and previous exposure to everolimus, which has been associated with cardiac dysfunction [18].

Although patients with sarcoma did not experience ≥ 10% decline in LVEF, one patient with a prior diagnosis of heart failure did have a cardiomyopathy event within six months of PLD cessation. This patient was able to receive three cycles of PLD prior to the cardiac event. It is uncertain if PLD was the sole contributing factor for this cardiac event, as this patient had a lower baseline LVEF of 50%-55% and was previously on pazopanib, another known agent that may cause cardiotoxicity [19,20]. The most common cardiotoxic agents used in the sarcoma group were pazopanib and ifosfamide.

Our findings contribute to the body of literature around this topic. One study analyzed 129 patients who received PLD with a median cumulative dose of 210 mg/m2 (range 25-300 mg/m2) in the metastatic BC setting [21]. Two or more cardiovascular risk factors were present in 70% of patients, including prior anthracycline therapy (60%), age > 60 years (57%), hypertension (39%), prior thoracic radiation (30%), and history of cardiac disease (27%). Five (4%) patients had some degree of cardiotoxicity, and only two severe cases of LVEF dysfunction were reported [21].

This study had several limitations, including a small sample size, a retrospective nature relying on accurate documentation, a single-center and observational study design, and the inconsistency of LVEF monitoring due to variations in clinical practice among providers. Further research is needed with a greater representation of each type of cancer and a larger sample size to further validate our findings. A multicenter retrospective study or meta-analysis study is likely necessary to obtain enough power to draw stronger conclusions.

## Conclusions

PLD administration in patients with sarcoma and BC and prior exposure to conventional doxorubicin was found to have an incidence of cardiotoxicity of 6%. Despite LVEF declines, only 2% of patients manifested clinical heart failure. At the time of this writing, there remain many lingering questions regarding the long-term safety of administration, and future studies or meta-analyses are likely necessary to confirm the safety of PLD use after conventional doxorubicin.
